# Annotations

**Published:** 1886-11-20

**Authors:** 


### Annotations.

Wanted, £50,000.

A sale of work in aid of the building fund of the Great Northern Central Hospital will take place on Thursday and Friday, December 2nd and 3rd, at the Northampton Hall, Highbury Corner. The sale will be held under the patronage of the Baroness Burdett-Coutts. The new Great Northern Central will be one of the largest hospitals which has been erected in London for many years, and will contain accommodation for a hundred and fifty in-patients. In consequence of the depression in trade, the committee find it extremely difficult to obtain the £50,000 required for the building. The people of North London, among whom the hospital is to be built, belonging chiefly to the trading classes, are seriously affected by the general depression, and are not taking up the matter in that liberal spirit which the occasion demands. The committee feel, however, that they must proceed with the work, because not only are the present hospital buildings in a ruinously dilapidated condition, but the number of beds in the hospital (thirty-three) is miserably inadequate to the wants of the vast population. The discrepancy between the needs of the district and the hospital accommodation is amazing and almost beyond belief. Such a district as North London should have one hospital bed for *every seven hundred* of its inhabitants. It really possesses one for *every twenty-five thousand*. The committee have boldly commenced building operations, in the hope that wealthy philanthropists, when they know the real facts of the case, will not leave them in the lurch. There is no doubt that funds are most urgently needed, and the hospital authorities deserve the utmost sympathy in their up-hill fight.

Cottage Hospitals.

The 27th Annual Report of the Cranleigh Village Hospital, the first cottage hospital ever established, has recently been issued. It must be profitable to recall the fact that the first cottage hospital was established to provide simplicity of domestic arrangements, the comfort of being within easy reach of relations and friends, and the quiet of a private room. To these comforts an attempt was to be made to cause a home feeling to prevail throughout the hospital, and so to add materially to its popularity in its own immediate district. The founders further desired to afford the patients a certain amount of liberty beyond that accorded to inmates of larger hospitals, and they have found that this has tended to aid the recovery of many of the patients. The only novel feature for providing income originally introduced was small payments by the patients. This feature is still present, and it has produced about one-sixth of the income each year. No money is received from workmen's subscriptions, congregational collections, legacies, endowment fund, or Hospital Sunday. With such modern means of "raising the wind" the original cottage hospital has no familiarity, although the larger hospitals, and the more recent cottage hospitals, depend to a very considerable extent upon the receipts from such sources for the wherewithal to defray the cost of maintenance. Nevertheless, twenty-seven years of useful existence, carried on in the same modest but adequate building, finds the Cranleigh Village Hospital with a balance in hand of £56, that is, £22 better off than it was a year ago. The lesson of the Cranleigh Village Hospital is that every village not within an easy distance of a hospital may provide itself with adequate accommodation of this character of the best and simplist kind, at a cost easily within the means of the inhabitants.

Lady Doctors and Nurses.

We desire to call attention to a letter which we have received from Philadelphia. It is written by Miss Alice Fisher, who is well known to many of those engaged in nursing in this country. She gives expression to the earnest desire which animates those who are responsible for the welfare of nurses in America, to provide adequate funds to secure pensions for those nurses who devote their lives to the work. She further points out, that good nurses are so abundant on this side of the Atlantic, that they find it hard to procure employment at a remunerative rate, and thinks they may with confidence, if possessed of a little money, decide to journey to the United States, where they will readily procure a large remuneration and much congenial work. Miss Fisher further states that she would not be surprised, seeing that the demand for women doctors in America is declining, if the nursing profession were to receive by-and-by many recruits from the ranks of lady doctors. We have made inquiries in America, and have held over Miss Fisher's letter, to enable us to do so, which go far to confirm her views on this point. It will be indeed a proud day for the nursing profession when the women doctors decide, if they ever do decide, that nursing provides a better means of livelihood for women than doctoring.

Teeth at the General and Special Hospitals.

The popularity of the National Dental Hospital, Great Portland Street, is causing it very considerable inconvenience. Every day, Sundays excepted, the waiting-room is filled with crowds of persons suffering with various degrees of toothache. Unfortunately, the crowds are too great for the space, which overflow into the hall and staircase—a state of things by no means pleasant, either to patients or operators. It is in contemplation to enlarge the premises, and as things are at present, we cannot but wish the managers success in their laudable efforts. It may, however, be asked what the general hospitals are doing with regard to the question of Practical Dentistry? Is there anything special in the character and constitution of the teeth, which causes them to be excluded from the friendly regard of the hospital-surgeon? A mere layman might ask why the eye and the ear, and the throat and the nose, are considered worthy of the attention of the regular surgeons at the recognised hospitals, whilst the poor teeth are relegated to less distinguished persons in less dignified institutions? It is difficult to exaggerate the value of sound and useful teeth. To say nothing of their importance as a feature of personal beauty, they play a most significant part in the work of sustaining the general economy of the body. Without teeth the orator gives utterance to a puerile lisp instead of to a well-defined vocable: without teeth the pangs of the dyspeptic are increased tenfold: without teeth the full, rounded contour of the matron's cheeks gives place to leathery hollows, and hard, bony mandibles: with bad teeth the sweet breath of the maiden is changed to an odour neither balmy nor delightful. For every reason, the general hospitals should bethink themselves, and repent of their unkindness to so pretty and so important an organ. Every general hospital should have a special and well-equipped department of dentistry, under the charge of a skilful and fully qualified surgeon; and this branch of the surgical art should be restored to an equality with its sister specialities,—a position from which it should never have been deposed.

